# Probing the Influence of Water on the Molecular Mobility of PVP/VA using Terahertz Spectroscopy

**DOI:** 10.1021/acs.molpharmaceut.5c00590

**Published:** 2025-09-08

**Authors:** Supawan Santitewagun, J. Axel Zeitler

**Affiliations:** Department of Chemical Engineering and Biotechnology, 2152 University of Cambridge, Cambridge CB3 0AS, U.K.

**Keywords:** amorphous solid dispersion, PVP/VA, terahertz time-domain spectroscopy, water, molecular mobility, differential scanning calorimetry

## Abstract

The presence of water significantly impacts the physical stability of amorphous solid dispersions (ASDs) by altering polymer molecular mobility. This study investigates the influence of low levels of absorbed water on the molecular dynamics and glass transition behavior of amorphous poly­(vinylpyrrolidone-*co*-vinyl acetate) (PVP/VA). Melt-quenched PVP/VA discs were conditioned at controlled relative humidities (RH 8.5 to 75%) and characterized using variable-temperature terahertz time-domain spectroscopy (THz-TDS, 80 to 420 K) and differential scanning calorimetry (DSC). DSC confirmed a significant plasticizing effect, with the glass transition temperature (*T*_g_) decreasing from 367 to 275 K with increasing hydration. THz-TDS absorption correlated linearly with water content at low temperatures and exhibited complex temperature dependence. Even the nominally dry sample displayed a high-temperature (ca. 350 K) absorption plateau, indicating tightly bound residual water, while the onset temperature of bulk water evaporation decreased systematically with increasing hydration, suggesting a greater proportion of loosely bound water. Empirical fitting (α­(*T*) = *AT* + *B* + *C*/*T*) revealed characteristic temperatures correlating with sub-*T*_g_ dynamics (*T*_12_) and *T*_g_ (*T*_23_), both sensitive to water content. Furthermore, the underlying polymer mobility rate was extracted from the THz data by accounting for evaporation; this analysis confirmed that water enhances mobility, potentially reaching a saturation limit at the higher hydration levels studied. This work highlights the capability of THz-TDS to provide detailed molecular-level insights into water–polymer interactions and dynamics, complementing thermal analysis and offering valuable information for assessing ASD stability.

## Graphical abstract

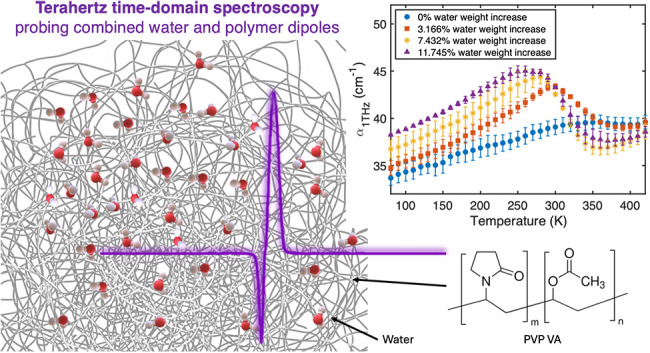

## Introduction

The poor aqueous solubility of many crystalline active pharmaceutical ingredients (APIs) presents a significant hurdle in drug development, often leading to limited dissolution, insufficient systemic exposure, and ultimately, reduced therapeutic efficacy.^1^ Formulating APIs in their amorphous form within polymeric matrices, creating amorphous solid dispersions (ASDs), is a widely adopted strategy to enhance solubility, dissolution rate, and bioavailability.^1,2^ However, the inherent thermodynamic instability of the amorphous form makes these systems prone to recrystallization back to the more stable crystalline state, compromising the formulation’s advantages.^3^ Polymeric excipients are crucial for stabilizing the amorphous API within an ASD, primarily by reducing molecular mobility and inhibiting nucleation and crystal growth.^4^ Nevertheless, selecting the most suitable polymer often involves empirical screening, partly due to an incomplete understanding of the specific drug-polymer interactions and the factors governing the physical stability of the dispersion.^5^

Poly­(vinylpyrrolidone)-vinyl acetate copolymer (PVP/VA), commonly known by its trade name Kollidon VA 64, is frequently employed in ASD formulations. It is synthesized by copolymerising hydrophilic N-vinylpyrrolidone (VP) and more hydrophobic vinyl acetate (VA), typically in a 60:40 weight ratio.^6^ Compared to its homopolymer precursor poly­(vinylpyrrolidone) (PVP), PVP/VA exhibits reduced hygroscopicity due to the presence of VA units while largely retaining the ability to stabilize amorphous drugs.^7,8^ Despite this reduced hygroscopicity, PVP/VA, like many pharmaceutical polymers, remains susceptible to moisture sorption from the ambient environment.^9^

Water uptake is a critical factor influencing the stability of ASDs. Absorbed water acts as a potent plasticizer, significantly increasing molecular mobility and reducing the glass transition temperature (*T*_g_) of the formulation.^10^ For instance, the *T*_g_ of amorphous indomethacin decreases by approximately 10 K for every 1% (w/w) increase in water content,^11^ although antiplasticization effects have occasionally been reported.^12^ Even seemingly negligible amounts of water, often present inherently or absorbed during storage, can profoundly impact the system’s dynamics.^13,14^ This increased mobility associated with water sorption can accelerate API recrystallization, promote phase separation between the drug and polymer, and potentially facilitate chemical degradation, thereby reducing the shelf life and efficacy of the product.^9,13,15^ Therefore, understanding the precise influence of water, particularly at low concentrations, on the molecular mobility of the polymer matrix is paramount for predicting and ensuring the long-term physical stability of ASDs.

Investigating molecular mobility and intermolecular interactions, especially hydrogen bonding perturbed by water, requires sensitive analytical techniques. Terahertz time-domain spectroscopy (THz-TDS) has emerged as a valuable noninvasive tool for characterizing pharmaceutical materials, offering unique sensitivity to molecular structure, intermolecular vibrations, and hydration states.^16,17^ While crystalline materials often exhibit sharp spectral features corresponding to phonon modes, amorphous systems display characteristic broad absorption profiles in the terahertz region.^18^ This broad absorption arises from the vibrational density of states (VDOS), reflecting the collective, disordered vibrational motions within the material.^19–22^ The position and shape of the VDOS are sensitive probes of intermolecular forces, including hydrogen bond strength and dynamics.^23^ Furthermore, THz-TDS is exceptionally sensitive to water due to water’s strong dipole moment, its picosecond relaxation times and extensive hydrogen-bonding capabilities, which interact strongly with terahertz radiation.^24,25^

This study leverages the sensitivity of THz-TDS to intermolecular interactions and molecular dynamics to investigate the influence of small amounts of absorbed water on the molecular mobility of the pharmaceutically relevant copolymer PVP/VA. By exposing PVP/VA samples to controlled humidity environments and performing temperature-dependent THz-TDS measurements, we aim to characterize changes in the VDOS as a function of both water content and temperature. These spectroscopic insights into molecular mobility are complemented and validated by differential scanning calorimetry (DSC) measurements of the glass transition temperature (*T*_g_), providing a comprehensive understanding of how low levels of hydration affect the physical properties and potential stability of this widely used pharmaceutical excipient.

## Materials and Methods

### Materials

Poly­(vinylpyrrolidone-*co*-vinyl acetate) (PVP/VA, Kollidon VA 64) was kindly provided by BASF (Ludwigshafen, Germany). Magnesium nitrate hexahydrate (Mg­(NO_3_)_2_·6H_2_O), sodium nitrite (NaNO_2_) and silica gel desiccant were purchased from Fisher Scientific Ltd. (Leicestershire, UK, analytical grade or higher) and sodium chloride (NaCl) was purchased from a local supermarket.

### Sample Preparation via Vacuum Compression Molding

Amorphous discs of PVP/VA were prepared using a vacuum compression molding device (MeltPrep GmbH, Graz, Austria). Approximately 120 mg of PVP/VA powder was placed in the device. The powder was heated under vacuum (target pressure <1 mbar) to 446 K, a temperature significantly above the polymer’s glass transition temperature (*T*_g_ ≈ 378 K), and held for 30 min to ensure complete melting and removal of residual moisture or entrapped air. The molten sample was subsequently cooled within the device under vacuum to room temperature over approximately 30 min, yielding transparent, bubble-free discs with a diameter of 13 mm and thicknesses ranging from 0.5 to 0.8 mm. To confirm that the PVP/VA maintained its expected amorphous nature and that no degradation or phase changes occurred during thermal processing, the visual appearance (clear discs) and subsequent DSC analysis (presence of *T*_g_, absence of melting endotherm) were examined. The precise thickness of each disc was measured using a digital micrometer (accuracy ± 0.001 mm) and recorded for calculating the absorption coefficient from THz-TDS data. Discs were prepared fresh and stored in a desiccator containing silica gel prior to humidity conditioning. All samples were analyzed within 4 days of preparation to minimize the potential effects of physical aging.

### Controlled Humidity Conditioning

To investigate the effect of water content on PVP/VA, prepared discs were conditioned under four different relative humidity (RH) environments at room temperature (approximately 294 K).

- **Low RH (Vacuum):** For the driest condition, samples were placed inside the THz-TDS cryostat sample chamber (Janis Research Company, LLC., Massachusetts), which was evacuated using a turbomolecular pump (Turbolab 90/250 i, Leybold GmbH, Germany) to a pressure of approximately 2 mbar. Samples were held under these conditions at 294 K overnight (>12 h) prior to THz measurement. The approximate RH under these conditions was estimated using the ratio of the chamber pressure to the saturation vapor pressure of water at 294 K (approximately 23.4 mbar^26^), yielding RH ≈ (2 mbar/23.4 mbar) × 100% ≈ 8.5%.
- **Controlled RH (Saturated Salt Solutions):** Three higher RH environments were established using saturated aqueous solutions of magnesium nitrate hexahydrate (54% RH), sodium nitrite (65% RH), and sodium chloride (75% RH), prepared according to standard methods.^27^ Each saturated solution was placed in the base of a separate sealed desiccator. The headspace RH was allowed to equilibrate for at least 24 h before introducing the PVP/VA discs (placed on a wire mesh platform above the solution). The RH inside each chamber was monitored using a calibrated digital thermo-hygrometer (Rapid Electronics Limited, Essex, UK; ± 3% RH) at 294 K.

PVP/VA discs were equilibrated in the respective salt solution chambers for 24 h prior to analysis, a time previously shown to be sufficient for moisture uptake saturation in similar systems.^28^ The mass of each disc was measured using an analytical balance (±0.1 mg) immediately before and after humidity conditioning to determine the water content gravimetrically.

### Differential Scanning Calorimetry (DSC)

The glass transition temperature (*T*_g_) of PVP/VA samples equilibrated under different humidity conditions was measured using a Discovery DSC2500 (TA Instruments, New Castle, Delaware). Samples (3–7 mg) were hermetically sealed in Tzero aluminum pans immediately after removal from their respective humidity environment to prevent moisture loss or gain. A heat–cool-heat cycle was employed under a nitrogen purge (50 mL min^–1^). Samples were first heated from room temperature (298 K) to 393 at 10 K min^–1^ to erase thermal history. Subsequently, they were cooled to 183 at 10 K min^–1^ and held isothermally for 3 min. Finally, a second heating scan was performed from 183 to 473 K at 10 K min^–1^. The *T*_g_ was determined from the second heating scan as the midpoint of the step change in reversing heat capacity, using the TRIOS software (v5.7.0.56, TA Instruments).

### Terahertz Time-Domain Spectroscopy (THz-TDS)

THz-TDS measurements were performed using a TeraPulse 4000 spectrometer (Teraview Ltd., Cambridge, UK) operating in transmission mode. Equilibrated PVP/VA discs were mounted in a continuous-flow cryostat (Janis Research Company, LLC., Massachusetts) cooled with liquid nitrogen and temperature-controlled using a Lakeshore 330 (Ohio) controller. The sample chamber was purged with dry nitrogen gas throughout the measurements to minimize atmospheric water vapor absorption. THz spectra were recorded over a temperature range from 80 to 420 K. The temperature was ramped in steps of 10 K, with a 10 min equilibration time allowed at each temperature set point before data acquisition. For each sample measurement, a corresponding reference measurement was taken under identical conditions (temperature, purge) with the empty cryostat (no sample in the beam path). Each spectrum represents the average of 1,000 waveforms, acquired over the frequency range 0.3–3.0 THz (10–100 cm^–1^) with a spectral resolution of approximately 0.95 cm^–1^.

The frequency-dependent absorption coefficient, α­(ν), was calculated using eq 1

1 $$\alpha \left(\nu \right)=-\frac{2}{d}\;\ln\left|\frac{E_{\mathrm{sam}}\left(\nu \right)}{E_{\mathrm{ref}}\left(\nu \right)}\right|$$

where *d* is the sample thickness (measured by micrometer), and *E*_sam_(ν) and *E*_ref_(ν) are the complex electric field amplitudes of the sample and reference THz pulses, respectively, obtained via Fourier transform of the measured time-domain waveforms. The temperature dependence of molecular mobility was initially assessed by plotting the absorption coefficient at 1 THz as a function of temperature.

## Results and Discussion

### Differential Scanning Calorimetry

Differential Scanning Calorimetry (DSC) was employed to determine the calorimetric glass transition temperature (*T*_g_) of PVP/VA samples equilibrated at different relative humidities (RH). The measured *T*_g_ can, in turn, provide an estimate of the sample’s moisture content, as water acts as a plasticizer, typically lowering the *T*_g_. Previous studies suggest an approximate 10 K decrease in *T*_g_ for every 1% (w/w) increase in water content for some amorphous systems.^11^ Therefore, a decrease in *T*_g_ was anticipated as the water uptake in the PVP/VA discs increased.

The measured *T*_g_ values for PVP/VA samples stored under different humidity conditions are presented in Table 1. The sample stored under the driest condition (vacuum, estimated 8.5% RH) was designated PVP/VA-1. Samples stored at progressively higher RH levels (54, 65, and 75%) were named PVP/VA-2, PVP/VA-3, and PVP/VA-4, respectively. The percentage weight increase upon humidification was measured gravimetrically using an analytical balance (Table 1). However, these gravimetric measurements exhibited significant variability between nominally identical discs, as indicated by the large standard deviations. This variability, potentially arising from subtle differences in disc handling or morphology affecting moisture sorption kinetics, made the precise determination of absolute water content challenging by direct weighing alone, particularly given the uncertainty in the baseline moisture of the PVP/VA-1 sample.

**Table 1:** *T*_g_ and Estimated Moisture Content of MeltPrep PVP/VA Stored in Different Humidity Conditions for 24 h

			weight increase (%)	est. moisture content (%)
sample	storage RH (%)	*T*_g_ (K)	(gravimetric)	(via Patel et al.^29^)
PVP/VA-1	8.5	367	0 (baseline)	0.9
PVP/VA-2	54	314	3.2 ± 2.4	11.2
PVP/VA-3	65	297	7.4 ± 4.0	18.2
PVP/VA-4	75	275	10.6 ± 2.4	28.5

Consequently, the moisture content was estimated by correlating the measured *T*_g_ values with published data from Patel et al.,^29^ who used dynamic vapor sorption (DVS) coupled with thermal analysis to establish a relationship between *T*_g_ and water content for PVP/VA. This correlative approach is expected to provide a more consistent estimate of the equilibrium water content achieved under each RH condition. The estimated moisture contents are included in Table 1. As expected, the measured *T*_g_ decreases significantly as the storage RH and estimated moisture content increase.

Figure 1 plots the measured *T*_g_ values against both the highly variable gravimetric weight increase (blue circles) and the moisture content estimated from the correlation with Patel et al. (red squares). Both representations illustrate the expected nonlinear plasticizing effect of water, where *T*_g_ decreases sharply with initial water uptake and then more gradually at higher water contents. The trend derived from the Patel et al. correlation appears smoother, likely reflecting equilibrium moisture content better than the variable gravimetric measurements. It is important to note potential differences between this study and Patel et al.; our samples were dense, compression-molded discs, whereas DVS studies often use powder samples, which may exhibit different sorption kinetics due to higher surface area. Furthermore, our *T*_g_ was measured by DSC on separate discs equilibrated ex-situ, while Patel et al. likely measured *T*_g_ in situ within the DVS instrument. Despite these differences, the correlation provides a reasonable estimate of water content for interpreting the THz-TDS results.

**Figure 1:**
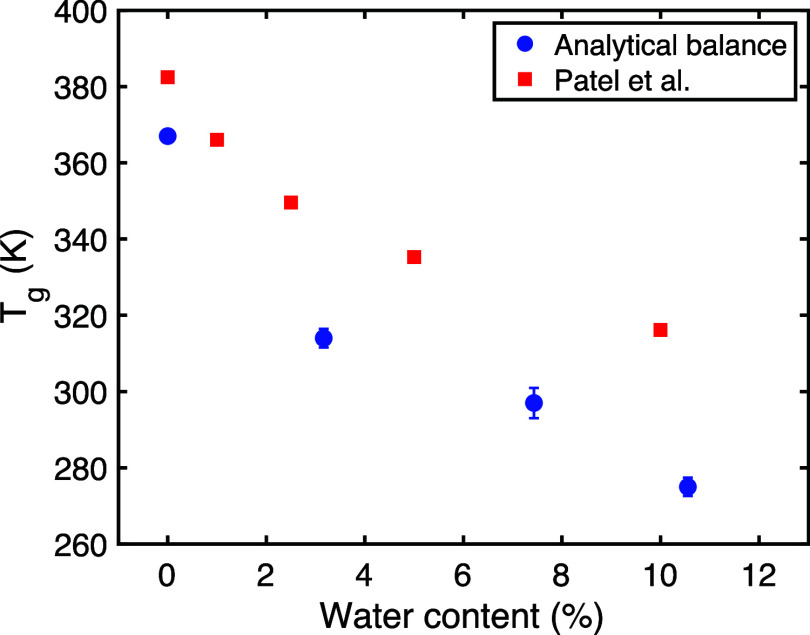
Glass transition temperature (*T*_g_) of PVP/VA versus water content. Blue circles represent *T*_g_ plotted against the gravimetrically measured weight increase (error bars show standard deviation, *n* = 3). Red squares represent the same *T*_g_ data plotted against the moisture content estimated via correlation with data from Patel et al.^29^

### THz-TDS of Controlled Humidity Samples

THz-TDS measurements were performed at 80 K to characterize the effect of water content on the spectroscopic properties while minimizing thermal energy and preventing water evaporation.^30^ The absorption coefficient, α, derived from these measurements reflects the dielectric loss associated with molecular dynamics, including vibrational modes and dipolar relaxations.^22^ In these hydrated samples, α arises from contributions from both the PVP/VA polymer and the sorbed water molecules, as well as their interactions. The frequency of 1 THz was selected for initial analysis due to the high signal-to-noise ratio in this region. Amorphous materials typically lack sharp spectral features at THz frequencies, exhibiting instead broad absorption profiles related to the vibrational density of states (VDOS).

The absorption coefficient at 80 K (Figure 2) provides insight into the relative water content. Plotting α­(1 THz) against the storage RH (Figure 2a) reveals a nonlinear relationship, consistent with the nonlinear water sorption isotherm typical for polymers. In contrast, plotting α­(1 THz) against the gravimetrically measured weight increase (Figure 2b) shows a clear linear correlation (*R* ^2^ ≈ 0.99). This strong linear relationship supports the use of THz absorption at low temperatures as a direct, albeit relative, measure of the water content within the discs, integrating contributions from polymer mobility, water mobility, and polymer–water interactions at this fixed temperature.

**Figure 2:**
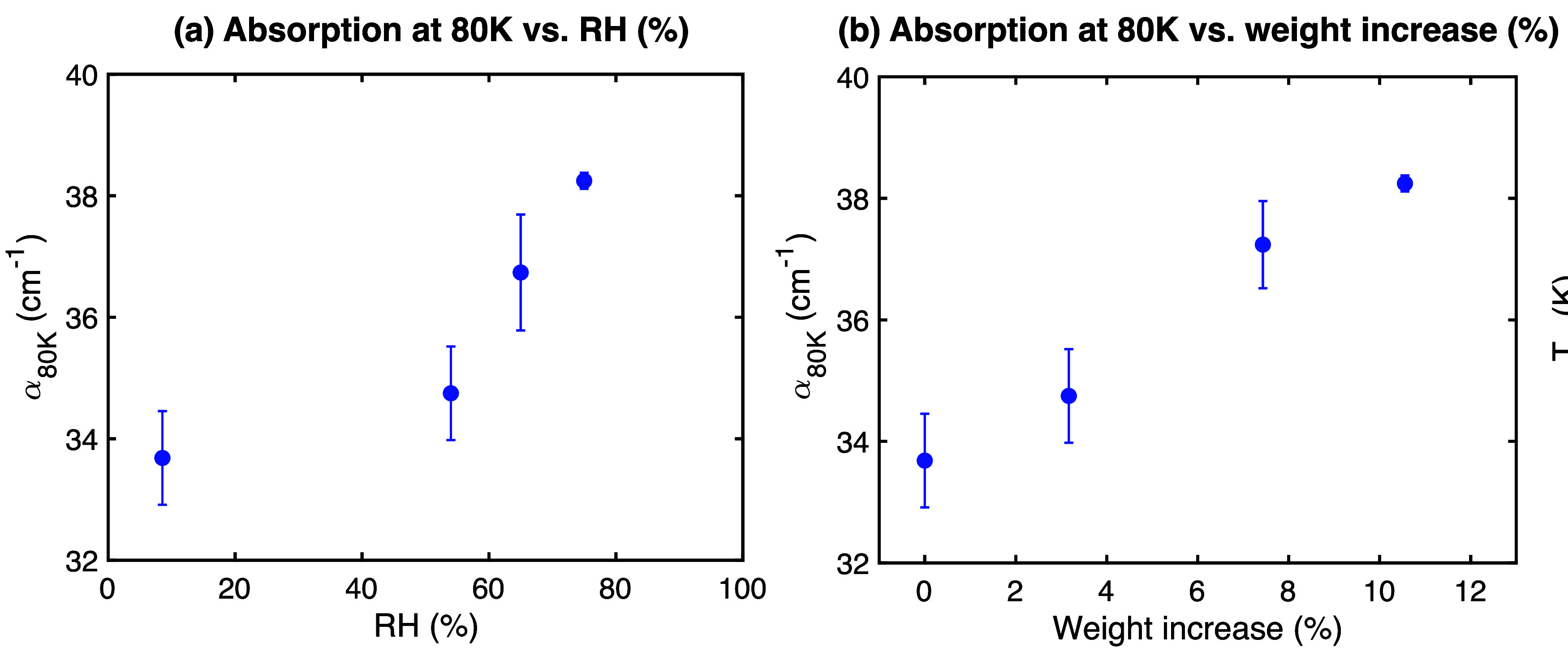
Absorption coefficient at 1 THz and 80 K for MeltPrep PVP/VA discs (thickness 0.5 mm to 0.8 mm) stored under different humidity conditions, plotted against (a) the storage RH value, and (b) the gravimetrically measured weight increase due to water uptake. Error bars show the standard error (*n* = 3) for each humidity level.

The full absorption spectra measured at 80 K are shown in Figure 3a. Some oscillations are present at low frequencies (<0.5 THz) and significant noise appears at higher frequencies (>2.5 THz), consistent with the reduced dynamic range of typical THz-TDS systems in these regions.^31^ Our wide span frequency-dependent analysis, shown in Supplementary Data, further revealed that signals above 1.4 THz showed increased fluctuations indicative of reduced SNR. Based on these spectral characteristics, a reliable window for quantitative analysis was identified between approximately 0.5 and 1.4 THz. Within this range, 1 THz consistently exhibited an excellent signal-to-noise ratio and demonstrated the most pronounced sensitivity to changes induced by moisture absorption in PVP/VA, making it ideal for our subsequent analyses. To better visualize the underlying trends despite the noise, the spectra were smoothed using a fourth-degree polynomial function (Figure 3b). It must be emphasized that this polynomial fit is purely empirical and not based on a physical model of the VDOS; it serves only to guide the eye regarding the shape of the broad absorption band.

**Figure 3:**
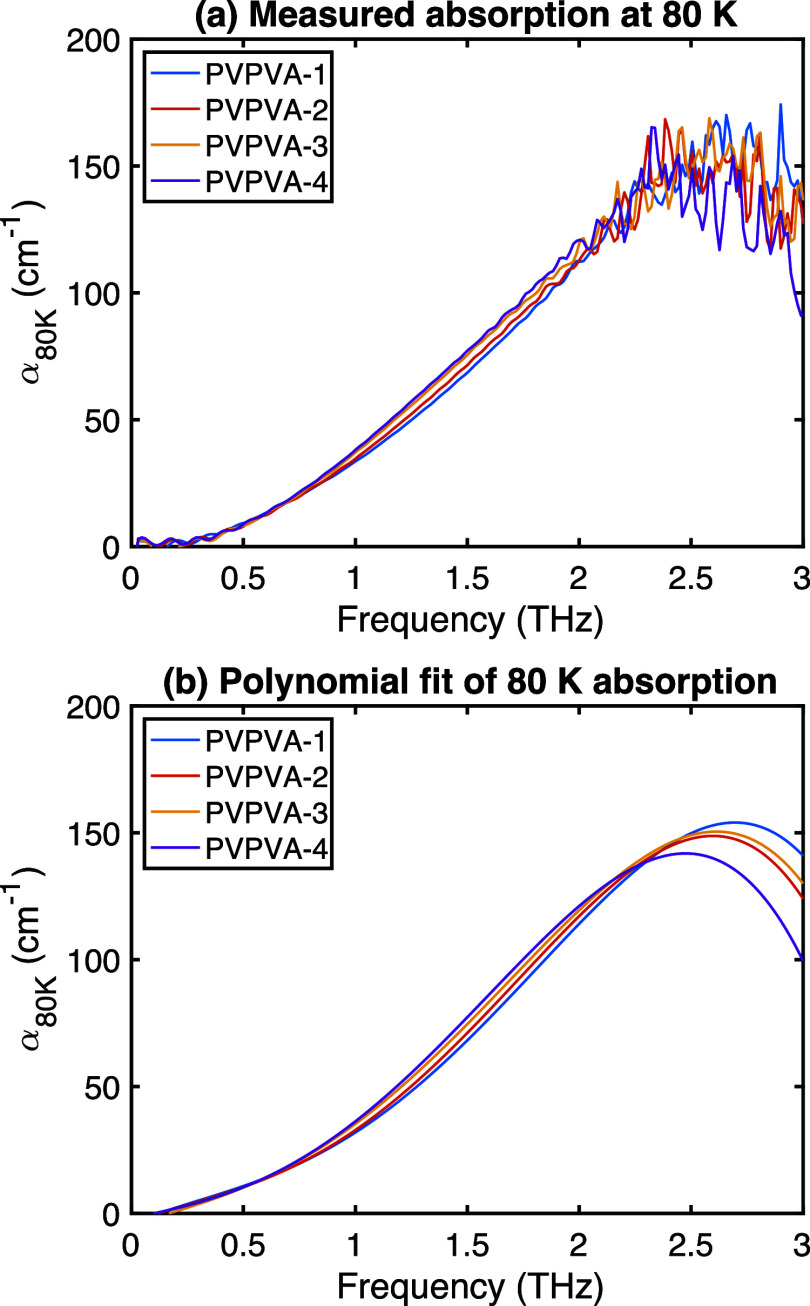
THz absorption spectra at 80 K for PVP/VA with different estimated water contents: (a) measured spectra, showing increased absorption with water content, and (b) corresponding fourth-degree polynomial fits used to smooth the data and visualize trends.

The smoothed spectra suggest two main effects of increasing water content at 80 K: (1) a general increase in absorption intensity across the measured range, particularly between 1 and 2 THz, consistent with the addition of absorbing water molecules; and (2) a subtle shift or broadening of the absorption peak toward lower frequencies (redshift) accompanied by a relative decrease in amplitude above 2 THz. Such redshifts in the THz VDOS at low temperatures are often associated with changes in intermolecular interactions, potentially indicating stronger or more numerous hydrogen bonds involving water molecules interacting with the VP units of the copolymer.^32^ This demonstrates the sensitivity of THz-TDS to hydration-induced changes in the polymer’s vibrational landscape, although confirmation of the high-frequency behavior would require measurements with broader spectral bandwidth.

### Variable Temperature Measurement

Variable temperature THz-TDS measurements were conducted from 80 to 420 K to investigate the influence of water on the temperature-dependent molecular dynamics of PVP/VA. The absorption coefficient at 1 THz, α­(1 THz), is plotted against temperature for the four samples in Figure 4.

**Figure 4:**
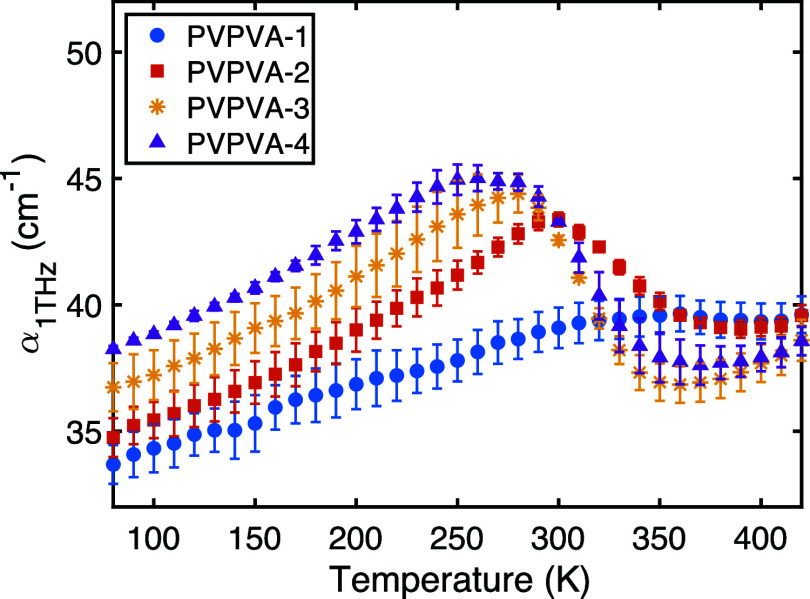
Temperature dependence of the absorption coefficient at 1 THz for PVP/VA samples equilibrated at different RH levels: PVP/VA-1 (8.5% RH, blue), PVP/VA-2 (54% RH, red), PVP/VA-3 (65% RH, yellow), PVP/VA-4 (75% RH, purple). Error bars represent standard error (*n* = 3).

Generally, THz absorption is expected to increase with temperature due to increased thermal energy facilitating molecular motions (vibrations, librations, relaxations) that couple to the THz field.^33^ This behavior is observed for all samples at lower temperatures. For the driest sample, PVP/VA-1 (blue curve), α­(1 THz) increases steadily until approximately 350 K, where a slight plateau or decrease occurs. This subtle feature might indicate the evaporation of trace amounts of very tightly bound water molecules, not removed by the initial vacuum conditioning. It is also conceivable, albeit not in the scope of the present study, that a subtle, water-induced degradation of the polymer, perhaps at the N-vinylpyrrolidone amine, forms new hydrogen bond donors during high humidity storage that interact with existing carbonyl acceptors, further restricting molecular mobility.

In a study by Heugen et al. using terahertz spectroscopy to investigate the dynamics of water around solute molecules, they found that water molecules in close proximity to solute surfaces (becoming more tightly bound) exhibit slower dynamics due to hydrogen bond rearrangements.^34^ Similarly, Charkhesht et al.’s study on protein–water interactions using megahertz-to-terahertz dielectric spectroscopy demonstrated that water molecules bound to hydrophilic protein surfaces display slower dipole relaxation compared to bulk or loosely bound water molecules due to stronger hydrogen bonding.^35^ The observation of the lowest terahertz absorption and a plateau around 350 K for our driest sample PVP/VA-1 could therefore suggest that the residual water molecules are tightly bound within the polymer matrix. Once sufficient thermal energy is provided, these tightly bound water molecules overcome the binding forces and evaporate.

For the hydrated samples (PVP/VA-2, PVP/VA-3, PVP/VA-4), the presence of larger amounts of water significantly increases the overall absorption at low temperatures, consistent with the 80 K data. As temperature increases, α­(1 THz) initially rises but then exhibits a distinct peak or broad maximum, followed by a significant decrease. This decrease is attributed to the evaporation of the sorbed water from the discs under the nitrogen purge within the cryostat. The temperature at which evaporation becomes dominant (indicated by the peak/onset of decrease) systematically shifts to lower values as the initial water content increases: approximately 300 K for PVP/VA-2, 280 K for PVP/VA-3, and 260 K for PVP/VA-4. This suggests that water sorbed at higher RH levels is, on average, less tightly bound or has easier diffusion pathways out of the polymer matrix compared to the residual water in the driest sample. This is likely because the initial water molecules occupy high-affinity hydrogen bonding sites on the polymer, whereas subsequent molecules at higher RH are more loosely bound via weaker, water–water interactions, allowing them to evaporate more readily.

At temperatures above the main evaporation phase (approximately >360 K), the absorption for PVP/VA-2 begins to increase again, reflecting the intrinsic mobility increase of the now largely dry polymer, eventually merging with the curve for PVP/VA-1. For PVP/VA-3 and PVP/VA-4, the absorption also increases again at high temperatures but remains below that of PVP/VA-1. This discrepancy at high temperatures for the initially most hydrated samples might be attributed to physical changes in the disc induced by the significant water loss during the scan, such as the formation of microcracks or voids, which could increase scattering of the THz beam and lead to an apparent reduction in the measured absorption coefficie The slope of the absorption versus temperature curve (dα/d*T*, with units of cm^–1^ K^–1^) in the low-temperature region (e.g., below 250 K, before significant evaporation) also appears to increase with water content. This suggests that water not only increases the baseline absorption but also enhances the temperature sensitivity of the molecular dynamics probed by terahertz radiation in this range.

### Fitting of 1 THz Data

While early studies on simple amorphous drugs often showed a quasi-linear increase in terahertz absorption with temperature below *T*_g_,^36^ potentially linking changes in slope to sub-*T*_g_ relaxations (around 0.6–0.7 *T*_g_),^36,37^ more recent work on complex systems like lyophilized proteins^25^ and, as observed here for hydrated PVP/VA, demonstrates significant nonlinearity. The curves in Figure 4 exhibit distinct regions of changing curvature (convex at low *T*, concave during evaporation). The lack of linearity may be due to the distribution of conformational states in the macromolecular samples compared to the more limited conformational configuration in small organic molecules.

To quantify these nonlinear changes, the empirical fitting function proposed by Kölbel et al.^25^ was applied to distinct temperature regions of the α­(1 THz) vs *T* data

2 $$\alpha \;\left(1\;\mathrm{THz},T\right)=\mathrm{AT}+B+\frac{C}{T}$$

Here, *A* represents a linear temperature dependence (cm^–1^ K^–1^), *B* is a constant offset term (cm^–1^), and *C* quantifies the degree of curvature via a reciprocal temperature dependence (K cm^–1^). While this function is empirical, the reciprocal *C* term, which quantifies the degree of curvature, was found to be particularly sensitive to water content. Its significance appears to grow with system complexity, suggesting it may reflect changes in the nature of water–polymer interactions and dynamics. The data for each sample was divided into segments based on visual changes in curvature, and each segment was fitted using eq 2. The resulting fits and residuals are shown in Figure 5. The low-temperature region (blue line), intermediate region (red line, often encompassing evaporation), and high-temperature region (purple line, postevaporation) are indicated.

**Figure 5:**
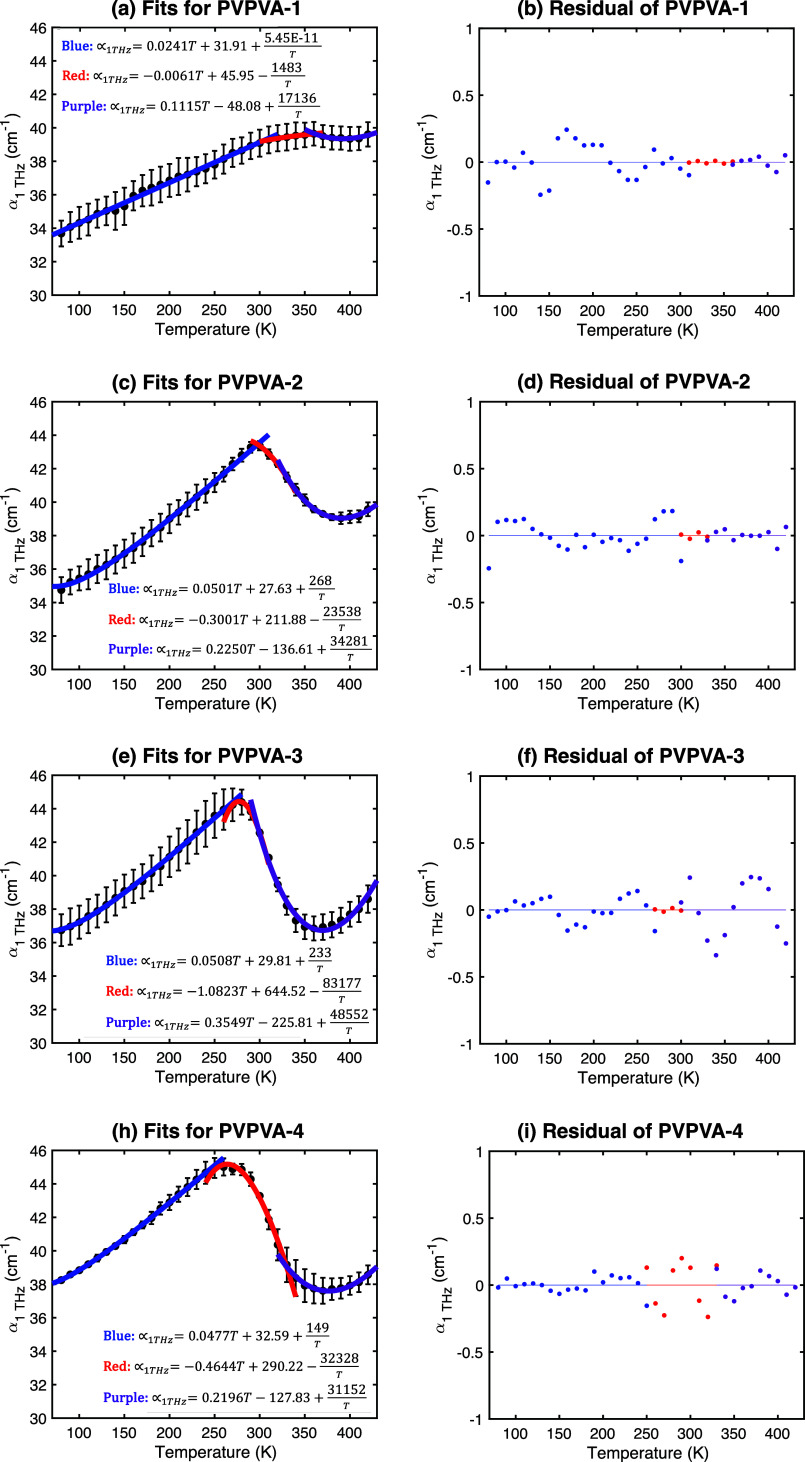
Fits (lines) to the experimental α­(1 THz) data (points) for PVP/VA samples using eq 2 applied to distinct temperature regions (Low T: blue; Intermediate T: red; High T: purple). Residuals of the fits are shown below each plot.

The fits generally capture the trends well, with low residuals. The intersection points between the fitted regions define characteristic temperatures, *T*_12_ (low to intermediate) and *T*_23_ (intermediate to high), summarized in Table 2 alongside the DSC *T*_g_ values.

**Table 2:** Characteristic Temperatures Derived from Fitting THz Data (Fig. 5) Compared with Calorimetric *T*_g_ (from Table 1)­^a^

sample	*T*_12_ (K)	*T*_23_ (K)	*T*_g_ (K, DSC)
PVP/VA-1	303	361	367
PVP/VA-2	298	332	314
PVP/VA-3	271	298	297
PVP/VA-4	252	330*	275

a *T*_12_ is the intersection between low and intermediate temperature fits; *T*_23_ is the intersection between intermediate and high temperature fits. The asterisk indicates a potential outlier.

As moisture content increases, *T*_12_ decreases systematically from 303 to 252 K. *T*_23_ initially decreases from 361 to 298 K (PVP/VA-1 to PVP/VA-3) but then increases to 330 K for PVP/VA-4. This latter value appears anomalous and might be a fitting artifact or perhaps related to the hypothesized sample cracking influencing the high-temperature data for this sample. Interestingly, *T*_23_ shows reasonable agreement with the calorimetric *T*_g_ for samples 1 and 3, suggesting this intersection might probe dynamics related to the onset of large-scale segmental mobility, similar to *T*_g_ but potentially reflecting the faster time scale of THz spectroscopy. *T*_12_ occurs significantly below *T*_g_ and decreases with increasing water content, consistent with expectations for a sub-*T*_g_ relaxation process (like the Johari–Goldstein β-relaxation), to which THz spectroscopy is known to be sensitive (referred to as *T*_g,β_ in^20,37–44^ and *T*_g_^*^ in ref 22). The presence of water clearly complicates the interpretation, indicating that while the fitting model captures the trends, linking these intersection points definitively to specific relaxation processes requires further investigation.

Analysis of the fitting parameters for the low-temperature region (Table 3), relevant for typical storage conditions (*ca. T*_g_ – 50 K^45^), provides further insight.

**Table 3:** Parameters Derived from Fitting the Low-temperature Region (ca. 80 to 250 K) of *α*(1 THz) Data Using *α* = *AT* + *B* + *C*/*T*

			
sample	linear term, *A* (cm^–1^ K^–1^)	offset term, *B* (cm^–1^)	reciprocal term, *C* (K cm^–1^)
PVP/VA-1	0.0241	31.91	0.0^a^
PVP/VA-2	0.0501	27.63	268
PVP/VA-3	0.0508	29.81	233
PVP/VA-4	0.0477	32.59	149

a Value for PVP/VA-1 negligible/zero within fitting uncertainty.

For the driest sample (PVP/VA-1), the reciprocal term *C* is negligible (effectively zero within fitting uncertainty), indicating an almost linear increase in absorption with temperature (*A* = 0.0241 cm^–1^ K^–1^). For the hydrated samples, *C* becomes significant and positive, introducing convex curvature. This may also strengthen the hypothesis that the curvature is related to the interaction of the mobile water molecules with the broader distribution in conformations that are present in macromolecules, and the nonlinear increase in absorption could be related to the increased ability of the water dipole to sample multiple adjacent hydrogen bonding sites in analogy to the onset of dihedral angle conformations observed in sorbitol.^41^ The linear term *A* roughly doubles for the hydrated samples (around 0.05 cm^–1^ K^–1^) compared to the dry sample but does not change systematically between PVP/VA-2, 3, and 4. This suggests water significantly increases the temperature dependence of mobility (higher *A*), possibly reflecting contributions from water dynamics or water-plasticized polymer motions. The offset term *B* shows a roughly increasing trend for the hydrated samples (PVP/VA-2 to 4), potentially correlating with the increasing baseline water content probed at low temperatures (cf. Figure 2b), although PVP/VA-1 deviates, likely because its behavior is dominated by the linear term without the significant *C*/*T* contribution affecting the conceptual intercept *B*, due to the relatively small number of water molecules present in this sample.

### Estimation of Mobility and Evaporation Rates

To further explore the dynamics, particularly separating the influence of water evaporation from the underlying polymer mobility changes at higher temperatures, an analysis based on the natural logarithm of the absorption coefficient, ln­(α), versus temperature was performed. Assuming the decrease in ln­(α) during the evaporation phase follows pseudo-first-order kinetics related to the Arrhenius law,^46^ the slope of this region can provide an estimate of the evaporation rate constant (with respect to temperature). The heating protocol involved 10 K steps with 10 min equilibration, resulting in an effective ramp rate influencing the observed kinetics.

A key assumption in this analysis is that the total measured absorption arises from contributions related to both the polymer matrix and the sorbed water, and that their effects on ln­(α) are approximately additive [i.e., ln­(α_total_) ≈ ln­(α_polymer_) + ln­(α_water_effect_)]. It is further assumed that the negative slope observed during evaporation predominantly reflects the removal of the water contribution (change in ln­(α_water effect_)). This linear evaporation trend is then subtracted from the overall ln­(α) vs *T* data in an attempt to isolate the underlying temperature dependence associated primarily with the polymer matrix mobility. The slope of the resulting “corrected” ln­(α) vs *T* plot is termed the “Mobility Rate” (*k*_mob_, in K^–1^), representing the temperature coefficient of the corrected logarithmic absorption. This approach is necessarily a simplification, as the underlying polymer dynamics may also contribute nonlinearly, but it aims to qualitatively decouple the two dominant processes observed.

Figure 6 shows plots of ln­(α) versus *T*. A linear region with a negative slope, corresponding to the dominant phase of water evaporation, was identified visually for each sample (temperature ranges specified in figure caption correspond to visually clearest linear decline). The slope of this linear fit provides an estimate of the “evaporation rate” (*k*_evap_, in K^–1^).

**Figure 6:**
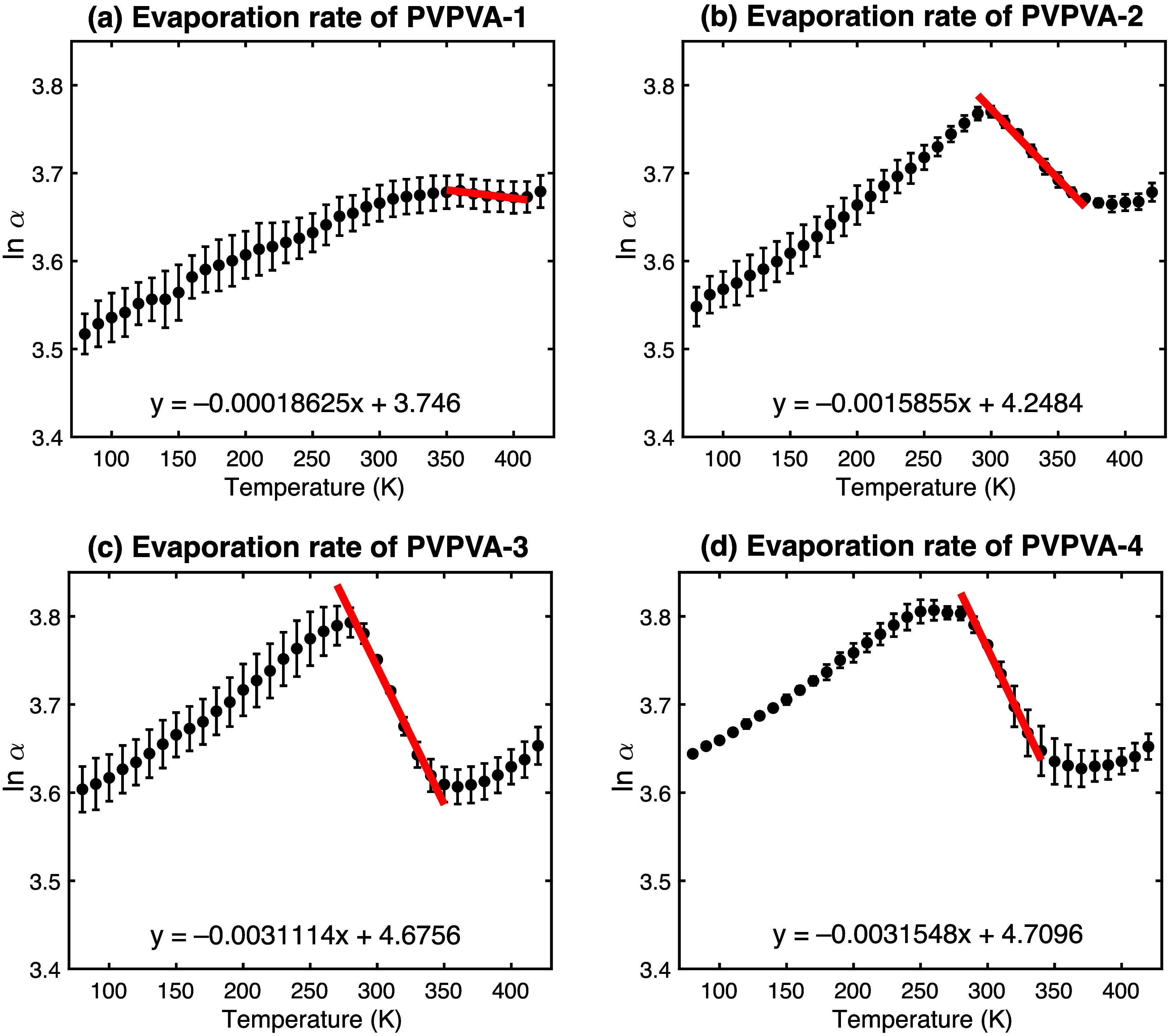
Natural logarithm of absorption coefficient at 1 THz versus temperature. The red lines indicate linear fits to the visually identified evaporation-dominant region (PVP/VA-1:360 to 400 K; −2:300 to 360 K; −3:280 to 340 K; −4:290 to 330 K), the slope of which estimates the evaporation rate constant (*k*_evap_).

The “corrected” ln­(α) vs *T* data, representing the estimated polymer mobility contribution, is shown in Figure 7. The slope of the linear fit applied to this data provides the estimated “Mobility Rate” (*k*_mob_).

**Figure 7:**
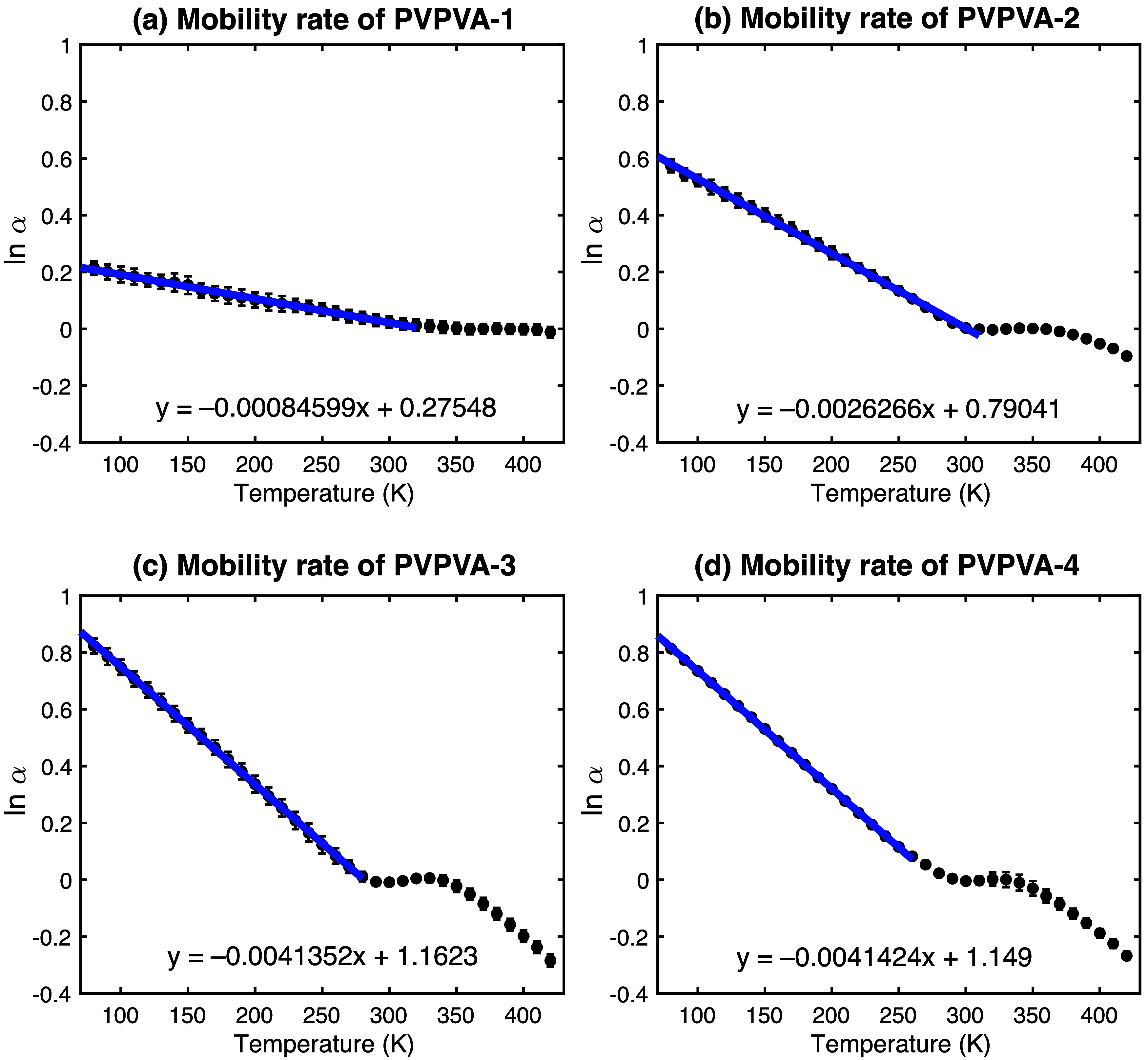
Estimated polymer mobility contribution (corrected ln­(α)) versus temperature, obtained by subtracting the linear evaporation trend (red lines in Figure 6) from the total ln­(α) data. The slope of the linear fit (blue lines) provides an estimate of the mobility rate constant (*k*_mob_).

The calculated evaporation and mobility rate constants are summarized in Table 4, using scientific notation for clarity.

**Table 4:** Estimated Evaporation Rate Constants (*k*_evap_) and Mobility Rate Constants (*k*_mob_) Derived from Linear Fits in Figures 6 and 7

sample	evaporation rate, *k*_evap_ (10^–5^ K^–1^)	mobility rate, *k*_mob_ (10^–5^ K^–1^)
PVP/VA-1	1.86 × 10^1^	8.46 × 10^1^
PVP/VA-2	1.59 × 10^2^	2.63 × 10^2^
PVP/VA-3	3.11 × 10^2^	4.14 × 10^2^
PVP/VA-4	3.15 × 10^2^	4.14 × 10^2^

The estimated evaporation rate (*k*_evap_) increases significantly with initial water content from PVP/VA-1 to PVP/VA-3, consistent with water being less tightly bound or evaporating more readily from the more hydrated samples. The estimated mobility rate (*k*_mob_) also increases substantially with water content. PVP/VA-1 exhibits the lowest mobility rate (8.46 × 10^–4^ K^–1^), reflecting the slower increase in mobility with temperature for the drier polymer. The rate increases significantly for PVP/VA-2 and further for PVP/VA-3 and PVP/VA-4, which show similar high rates (ca. 4.14 × 10^–3^ K^–1^). This suggests that water markedly increases the temperature sensitivity of the polymer’s mobility, potentially reaching a saturation level at the higher water contents studied.

Plotting these rates against the estimated water content (Figure 8) visually confirms the strong influence of hydration. While based on simplified assumptions, this analysis provides quantitative estimates demonstrating that water not only increases the baseline terahertz absorption but also significantly enhances the rate at which molecular mobility increases with temperature in PVP/VA, a key factor influencing the physical stability of amorphous formulations.

**Figure 8:**
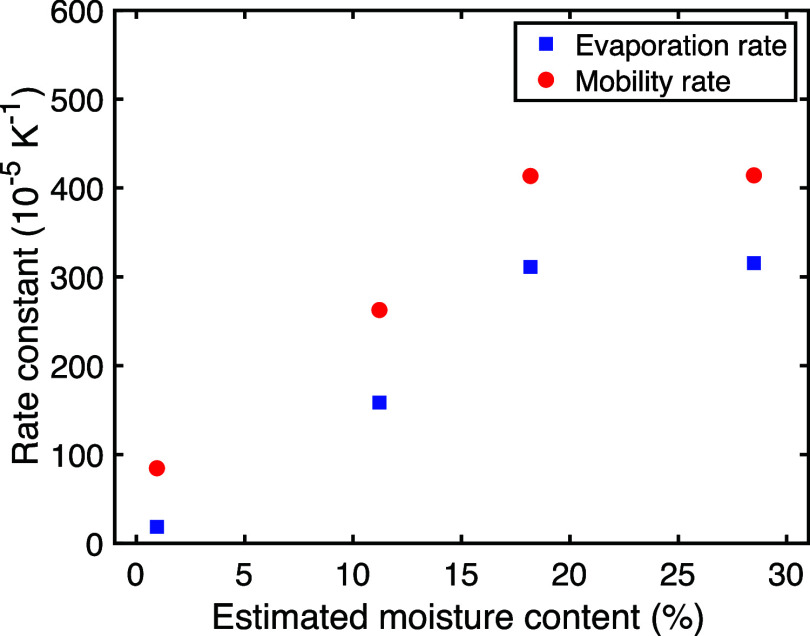
Estimated evaporation rate constants (*k*_evap_, squares) and mobility rate constants (*k*_mob_, circles) plotted against the estimated moisture content (from Table 1) demonstrates the strong dependence of both rates on the hydration level of PVP/VA.

## Conclusion

This study demonstrates the profound influence of low levels of absorbed water on the molecular mobility of the pharmaceutically relevant copolymer PVP/VA, utilizing a combination of terahertz time-domain spectroscopy (THz-TDS) and differential scanning calorimetry (DSC).

The absorption of water acts as a potent plasticizer, significantly depressing the *T*_g_ of PVP/VA as confirmed by DSC. THz-TDS corroborated this increased mobility, providing critical molecular-level insights into water content and interactions across broad temperature ranges, including the sub-*T*_g_ region inaccessible to conventional DSC. Quantitatively, the nonlinear temperature dependence of THz absorption in the hydrated samples was effectively modeled, yielding parameters that correlate with water content and dynamic transitions, including those below the calorimetric *T*_g_ that are crucial for long-term stability.

Overall, this work highlights the utility of THz-TDS as a powerful analytical tool for characterizing hydrated amorphous pharmaceutical systems. Understanding and quantifying how small amounts of water influence polymer dynamics is essential for predicting and controlling the physical stability of amorphous solid dispersions, thereby contributing significantly to advanced formulation characterization and stability assessment in molecular pharmaceutics.

## Supplementary Material



## Notes

DSC thermograms as well as stacked THz absorption spectra of PVP/VA-2 and the absorption spectra at 80 K for PVP/VA with different water content and their fit (PDF)
