# Supplementary material for: Probing the Influence of Water on the Molecular Mobility of PVP/VA using Terahertz Spectroscopy
Source: Mol Pharm. 2025 Sep 8;22(10):5928–38. doi: 10.1021/acs.molpharmaceut.5c00590 (PMC12505260; doi:10.1021/acs.molpharmaceut.5c00590)
Supplement: Supplementary file 1 [file mp5c00590_si_001.pdf]

Supporting Information: Probing the Influence of Water on the Molecular Mobility of PVP/VA using Terahertz Spectroscopy

Supawan Santitewagun and J. Axel Zeitler*

Department of Chemical Engineering and Biotechnology, University of Cambridge, Cambridge CB3 0AS, UK

E-mail: jaz22@cam.ac.uk

The supporting information contains DSC thermograms (Figures S1 to S5) as well as stacked THz absorption spectra of PVP/VA-2 (Figure S6) and the absorption spectra at 80 K for PVP/VA with different water content and their fit (Figure S7).

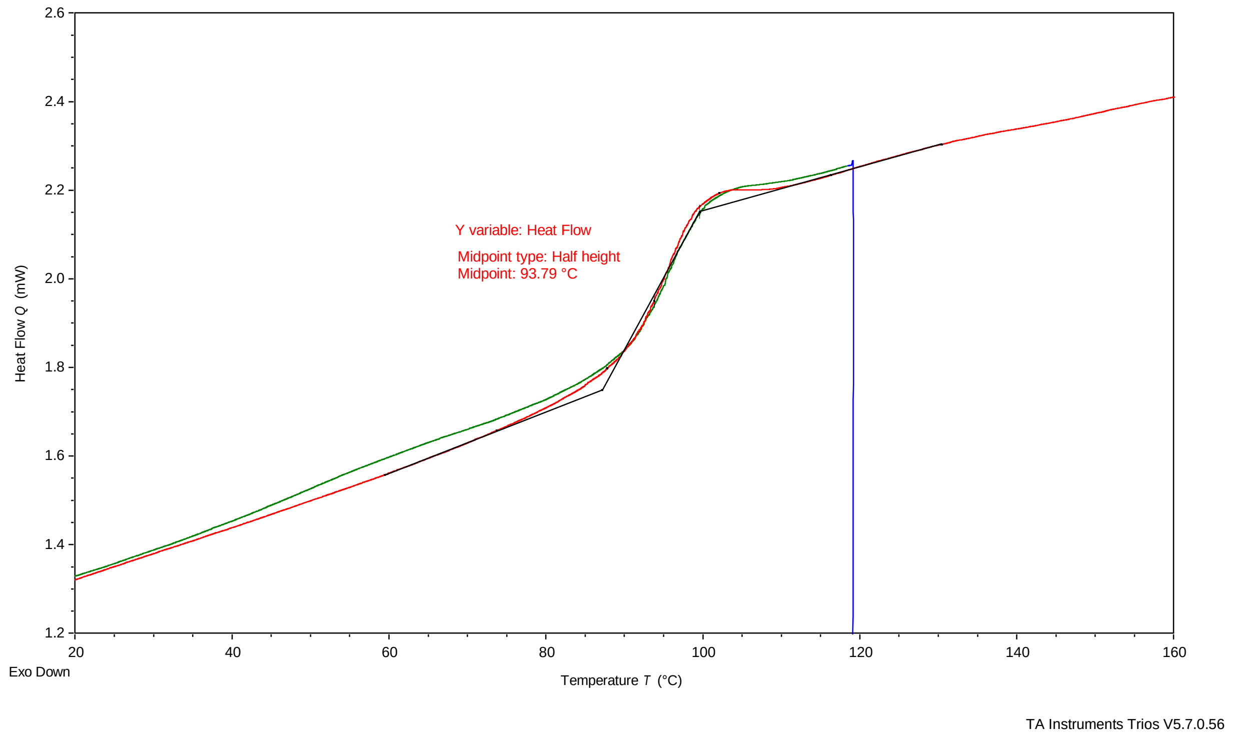

Figure S1: DSC thermogram of PVP/VA-1 (Sample 1) showing glass transition.

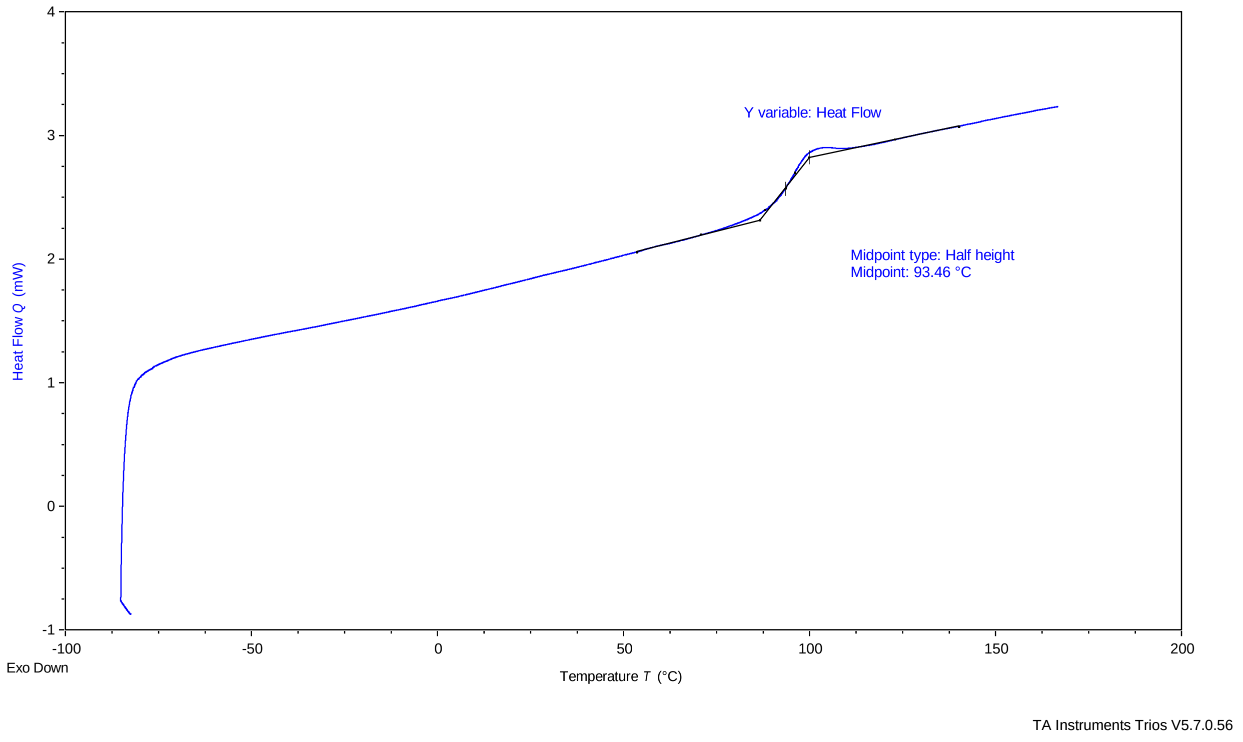

Figure S2: DSC thermogram of PVP/VA-1 (Sample 2) showing glass transition.

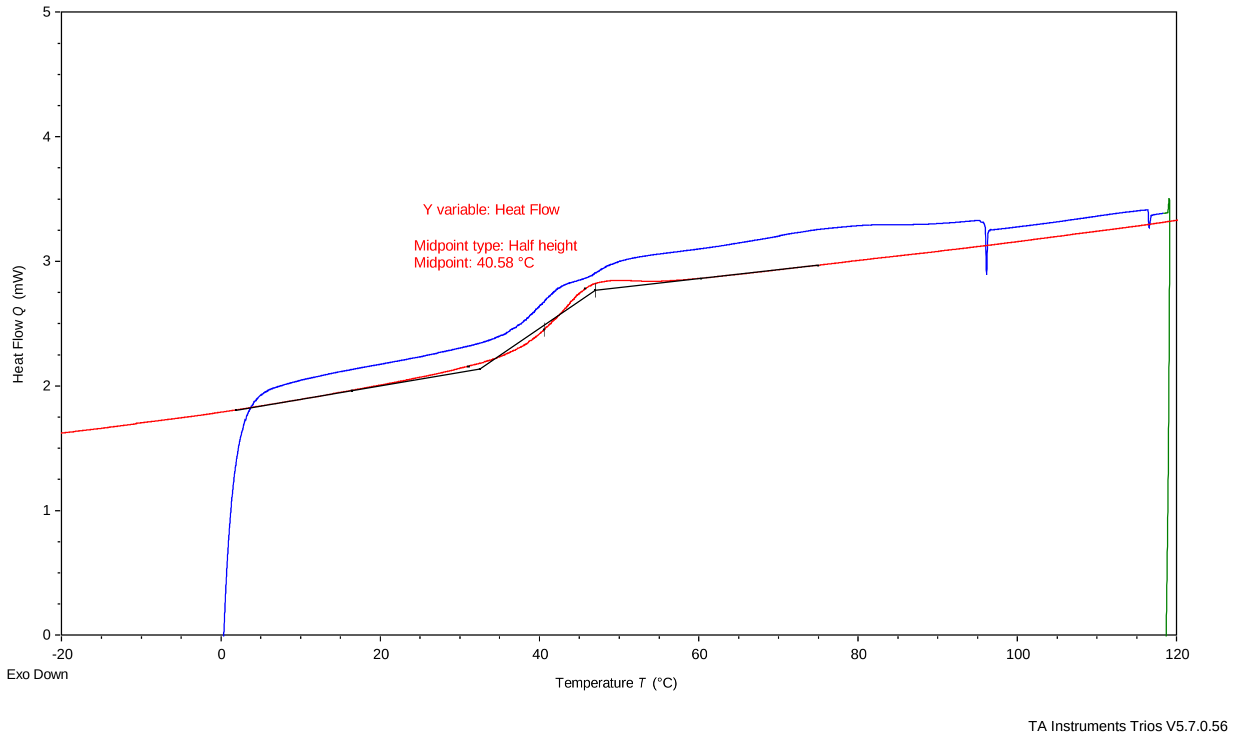

Figure S3: DSC thermogram of PVP/VA-2 showing glass transition.

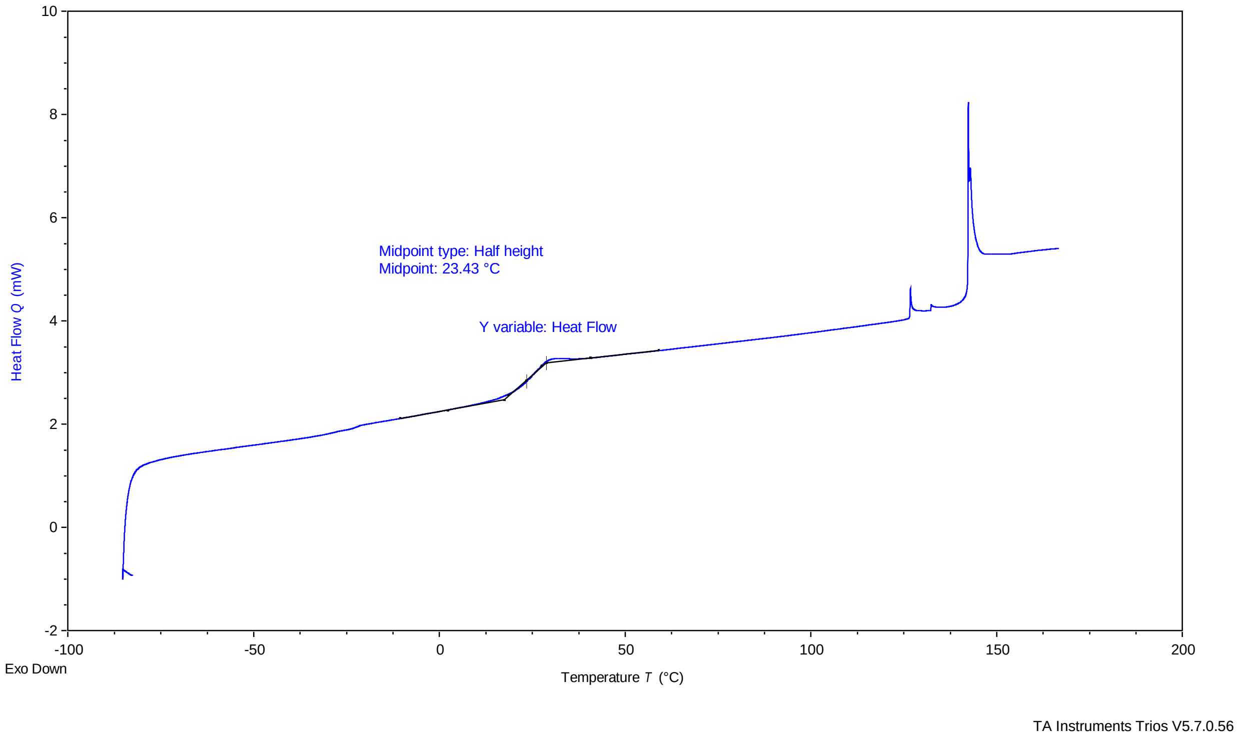

Figure S4: DSC thermogram of PVP/VA-3 showing glass transition.

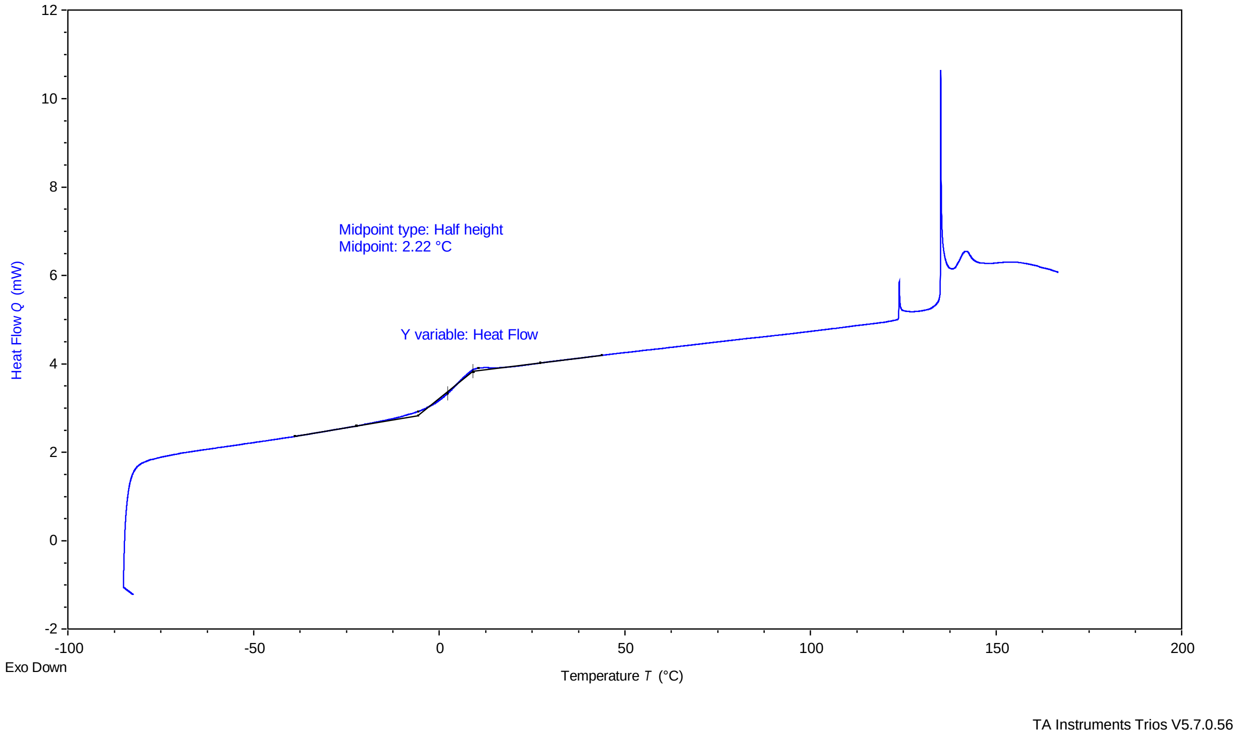

Figure S5: DSC thermogram of PVP/VA-4 showing glass transition.

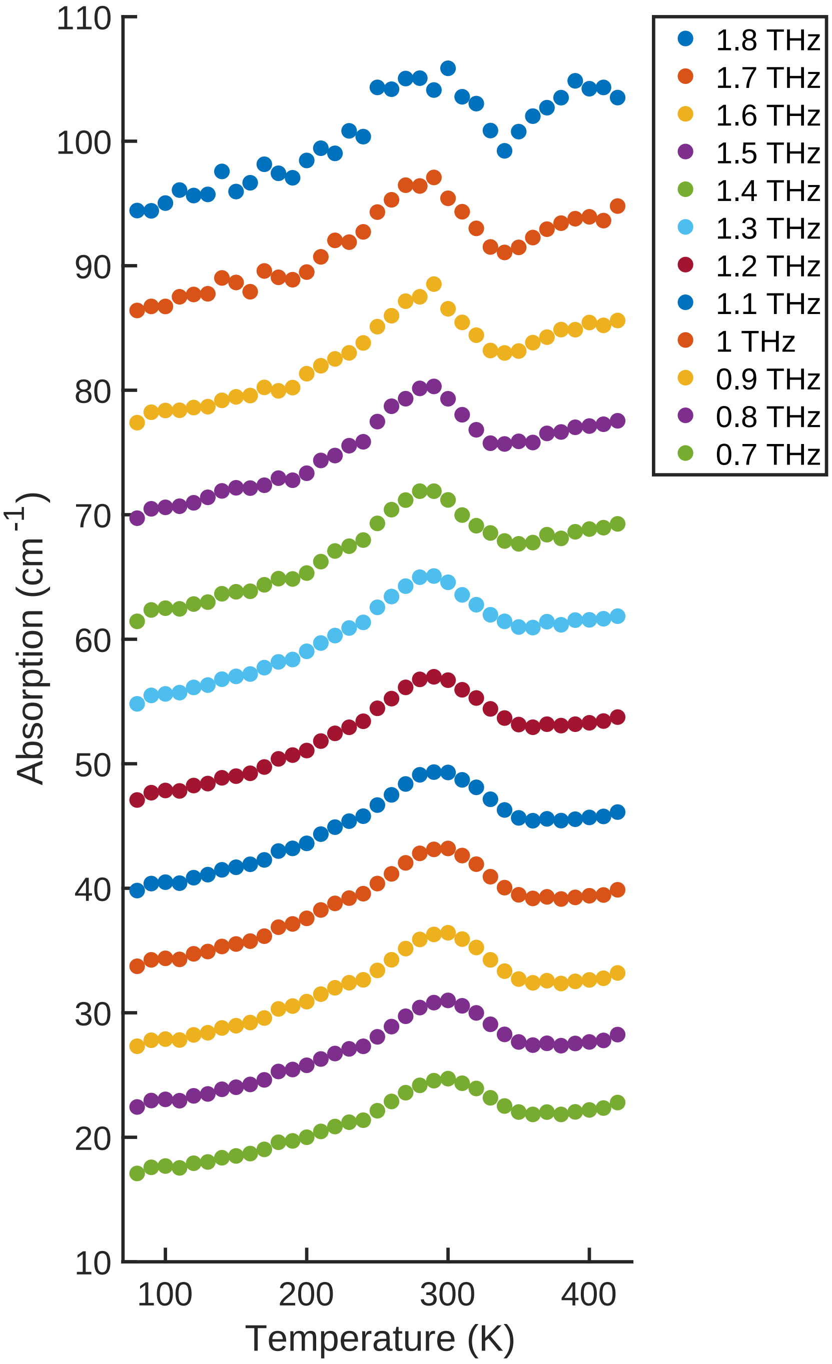

Figure S6: THz Absorption Spectra of PVP/VA-2 showing frequency dependence from 0.7 THz to 1.8 THz.

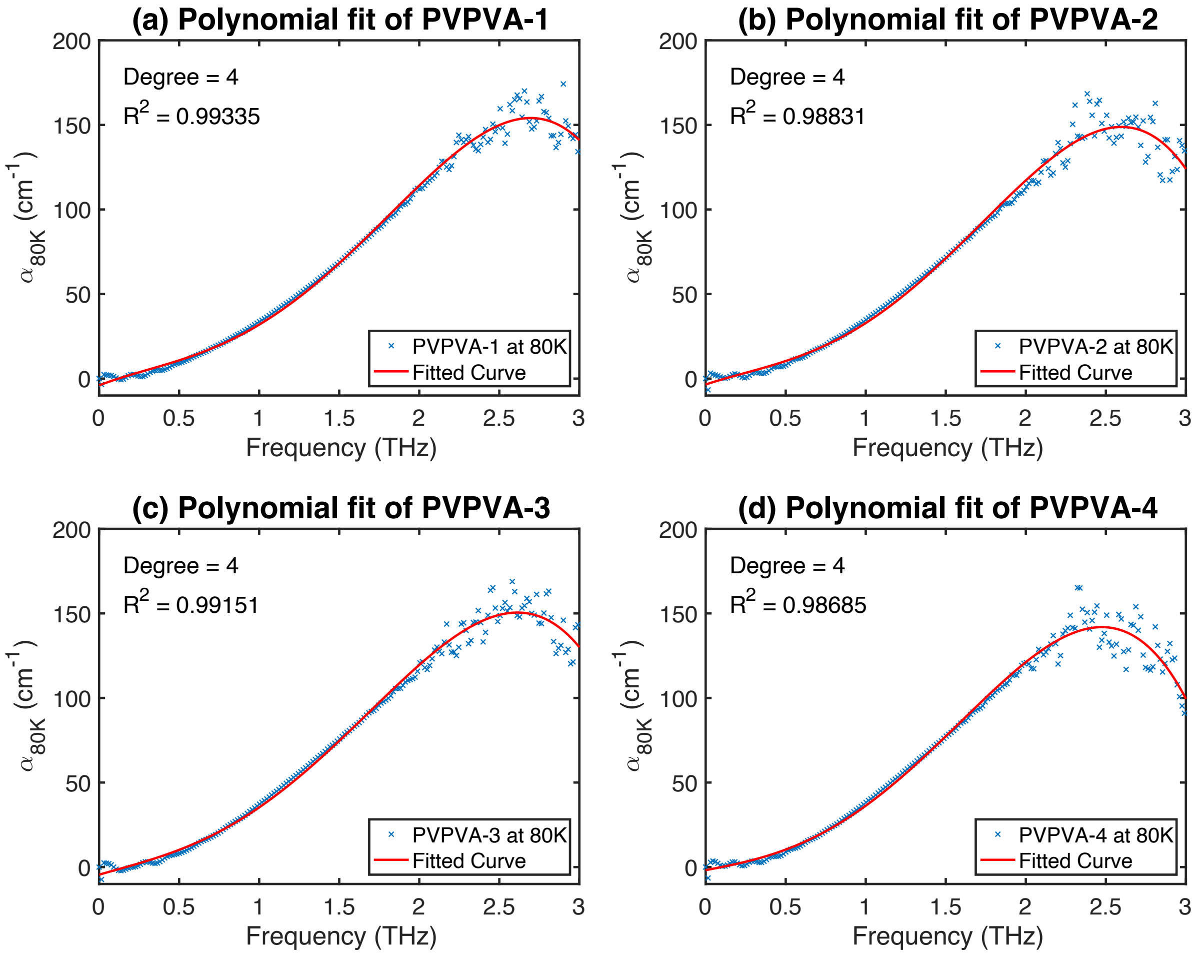

Figure S7: The absorption spectra at 80 K for PVP/VA with different water content were fitted with a 4th degree polynomial function.
